# Adverse cardiac remodeling is absent in patients with true controlled resistant hypertension

**DOI:** 10.1111/jch.14625

**Published:** 2023-04-03

**Authors:** Faris Matanes, Mohammed Siddiqui, Alejandro Velasco, Oleg Sharifov, Eric Kreps, Tanja Dudenbostel, Eric K Judd, Bin Zhang, Steven G Lloyd, Suzanne Oparil, David A Calhoun

**Affiliations:** ^1^ Vascular Biology and Hypertension Program University of Alabama at Birmingham Birmingham USA; ^2^ Jordan University of Science and Technology Irbid Jordan; ^3^ Department of Medicine University of Alabama at Birmingham Birmingham Alabama USA; ^4^ Division of nephrology University of Alabama at Birmingham Birmingham USA; ^5^ Cardiology Department Montefiore Medical Center Bronx New York USA; ^6^ Division of Biostatistics and Epidemiology Cincinnati Children's Hospital Medical Center Cincinnati Ohio USA; ^7^ Department of Pediatrics University of Cincinnati Cincinnati Ohio USA; ^8^ VA Medical Center Birmingham Alabama USA

**Keywords:** cardiac remodeling, diastolic function, left ventricular mass, masked uncontrolled hypertension, resistant hypertension

## Abstract

Resistant hypertension (RHTN), defined as blood pressure (BP) that is uncontrolled with ≥3 medications, including a long‐acting thiazide diuretic, also includes a subset with BP that is controlled with ≥4 medications, so‐called controlled RHTN. This resistance is attributed to intravascular volume excess. Patients with RHTN overall have a higher prevalence of left ventricular hypertrophy (LVH) and diastolic dysfunction compared to patients with non‐RHTN. We tested the hypothesis that patients with controlled RHTN due to the intravascular volume excess have higher left ventricular mass index (LVMI), higher prevalence of LVH, larger intracardiac volumes, and more diastolic dysfunction compared to patients with controlled non‐resistant hypertension (CHTN), defined as BP controlled with ≤3 anti‐hypertensive medications. Patients with controlled RHTN (n = 69) or CHTN (n = 63) who were treated at the University of Alabama at Birmingham were offered enrollment and underwent cardiac magnetic resonance imaging. Diastolic function was assessed by peak filling rate, time needed in diastole to recover 80% of stroke volume, E:A ratios and left atrial volume. LVMI was higher in patients with controlled RHTN (64.4 ± 22.5 vs 56.9 ± 11.5; *P* = .017). Intracardiac volumes were similar in both groups. Diastolic function parameters were not significantly different between groups. There were no significant differences in age, gender, race, body mass index, dyslipidemia between the two groups. The findings show that patients with controlled RHTN have higher LVMI, but comparable diastolic function to those of patients with CHTN.

## 1 INTRODUCTION

**FIGURE 1:**
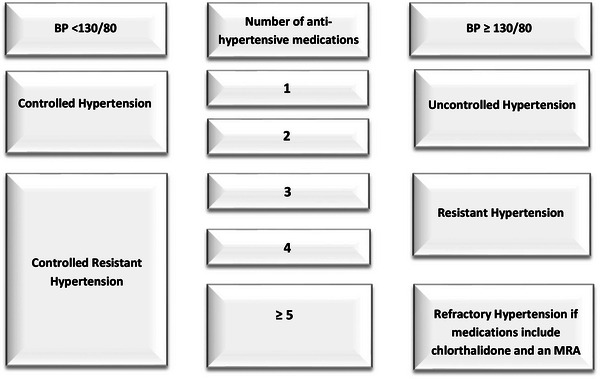
Phenotypes of hypertension. Abbreviations: BP, blood pressure; MRA, mineralocorticoid receptor antagonist

Resistant hypertension (RHTN) is defined as BP which is uncontrolled with ≥3 medications—commonly a long‐acting calcium channel blocker (CCB), a blocker of the renin‐angiotensin system [angiotensin‐converting enzyme (ACE) inhibitor or angiotensin receptor blocker (ARB)], and a diuretic, all prescribed at maximal or maximally tolerated doses and at the appropriate dosing frequency.^1^ A subset of RHTN, controlled RHTN, is BP that is controlled with ≥4 medications (Figure 1). The prevalence of RHTN among all patients with treated hypertension is 10–15%.^2^, ^3^, ^4^ Patients with either uncontrolled or controlled RHTN have a higher risk of developing cardiovascular (CV), neurological, and renal adverse outcomes than patients with non‐RHTN.^5^, ^6^ However, controlled RHTN patients have rarely been studied as a separate entity and thus the cardiac structure and function of these patients is not well characterized compared to patients with controlled non‐RHTN (CHTN).

We have previously demonstrated intravascular volume excess, as indicated by increased atrial natriuretic peptide (ANP) and B‐type natriuretic peptide (BNP) levels, in patients with RHTN compared to normotensives and patients with CHTN.^7^ Thus, intravascular volume excess appears to be a major pathophysiologic mechanism underlying resistance to anti‐hypertensive treatment. Since volume overload is a known risk factor for LVH,^8^ in this study, we tested the hypothesis that RHTN patients with controlled BP would have higher LV mass, higher prevalence of LVH, larger intracardiac volumes, and more diastolic dysfunction than those with CHTN.

Diastolic dysfunction is present in approximately 50% of patients with clinical congestive heart failure,^9^ and has been associated with worse CV outcomes.^10^, ^11^ Hypertension is one of the most prevalent risk factors for diastolic dysfunction.^12^, ^13^ BP control can improve diastolic function, and some ARBs and CCBs have been particularly helpful in achieving that goal.^14^, ^15^, ^16^ This study tested whether BP control can restore and/or preserve diastolic function in patients with controlled RHTN to levels similar to those in patients with CHTN.

## 2 METHODS

**FIGURE 2:**
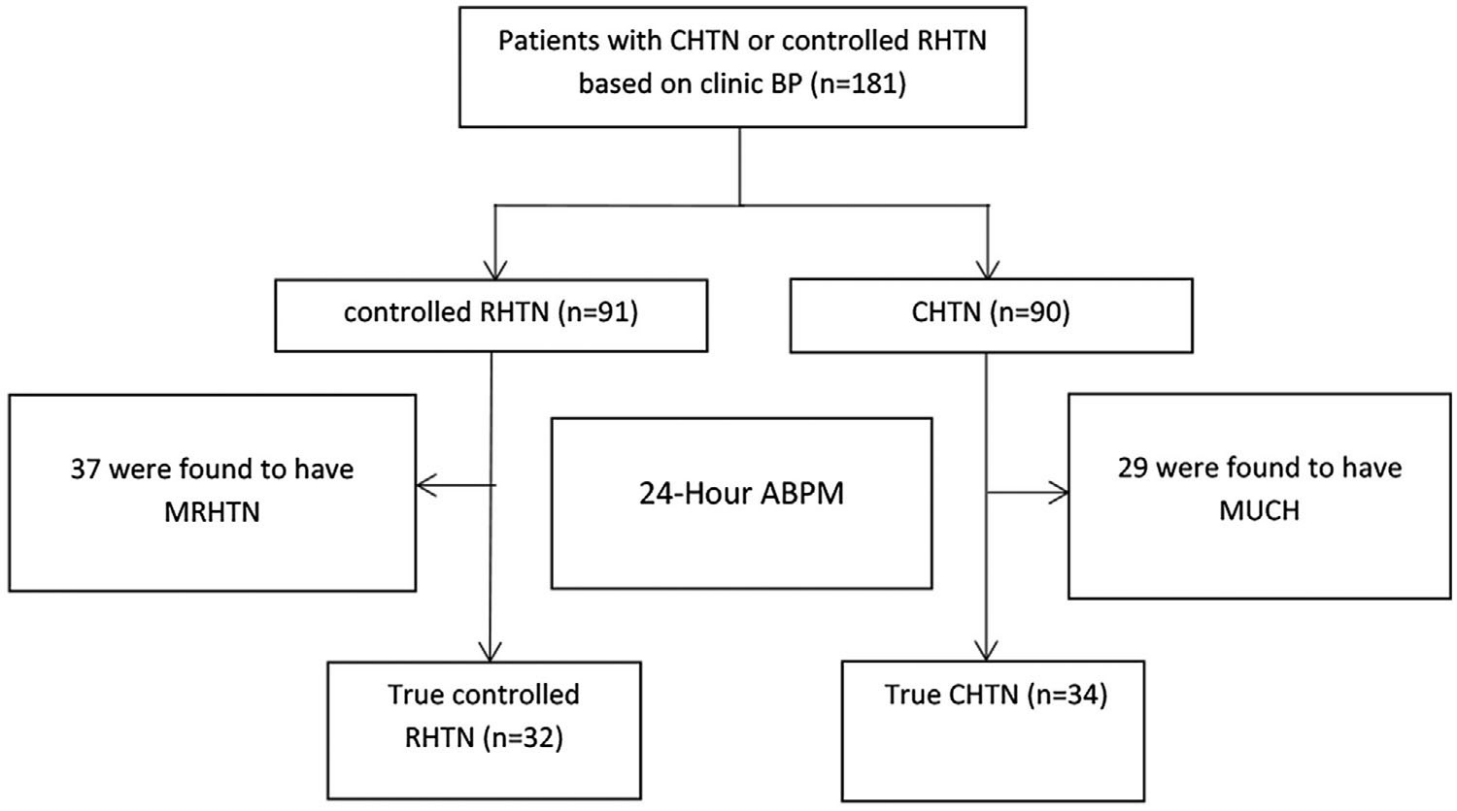
Patient selection process. Abbreviations: ABPM, ambulatory blood pressure monitoring; CHTN, controlled non‐resistant hypertension; HTN, hypertension; MRHTN, masked uncontrolled RHTN; MUCH, masked uncontrolled HTN; RHTN, resistant hypertension; Seven patients in the controlled RHTN group and nine in the CHTN group were excluded due to missing ABPM; Nine patients in the MRHTN, 10 in the MUCH, 6 in the true CRHTN, and 8 in the true CHTN were excluded due to missing CMR

Hypertensive patients were recruited from the University of Alabama at Birmingham (UAB) Hypertension Clinic. Controlled RHTN was defined as controlled office BP (≤135/85 mmHg) for at least three consecutive follow‐up visits on four or more medications, including a long‐acting thiazide diuretic (Figure 1). Patients then underwent 24‐h ambulatory blood pressure monitoring (ABPM) to assess BP control out of clinic (Figure 2). All patients with controlled RHTN underwent workup for hyperaldosteronism and pheochromocytoma. Patients with clinical signs suggestive of renal artery stenosis underwent assessment and were excluded. Other exclusion criteria included: chronic kidney disease stages 4 or 5 (estimated glomerular filtration rate < 30 mL/min/1.73 m^2^), non‐adherence to anti‐hypertensive medications based on self‐report, pregnancy, and nursing. The UAB Institutional Review Board approved the study, and written informed consent was obtained from all participants.

Automated office BP (AOBP) was measured using a BpTRU device (Coquitlam, British Columbia, Canada). Patients were allowed to rest in a sitting position with the back supported and the arm supported at heart level for 5 min.^17^ A proper cuff size was selected to ensure that the cuff bladder covered at least 80% of the patient's arm circumference.^18^ BP readings were taken unattended. This device automatically performs six BP readings, 1 min apart, and calculates the average of the last 5.^19^, ^20^ Office BP control was defined as ≤135/85 mmHg.^21^, ^22^ ABPM was measured using an automated noninvasive oscillometric device (Oscar 2; SunTech Medical Inc Morrisville, North Carolina, USA). BP was measured every 20 min while awake and every 30 min while asleep. BP control by ABPM was defined as average daytime BP ≤ 135/85 mmHg. Masked uncontrolled HTN was defined as BP that is controlled in three clinic visits (≤135/85 mmHg) but uncontrolled out of clinic (average daytime ABPM > 135/85 mmHg).^23^ Valid ABPM was defined as success in obtaining > 80% of BP measurements.^23^, ^24^ Non‐dipping nocturnal BP was defined as ratio of both mean nighttime systolic BP to daytime systolic BP and mean nighttime diastolic BP to daytime diastolic BP > .9.^25^ A separate analysis was carried out with patients with true controlled and masked uncontrolled HTN based on ABPM readings. Patient selection process resulted in 4 groups in total (Figure 2).

Cardiac magnetic resonance imaging (CMR) was performed to assess cardiac structure and function using a 1.5‐Tesla clinical scanner optimized for cardiac imaging (Signa, GE Healthcare). Standard short‐axis and two‐ and four‐chamber images were obtained. CAAS Flow was used to measure E and A wave filling velocities and CAAS MRV software (Pie Medical Imaging, Maastricht, the Netherlands) was used to measure left atrium (LA) volume, LV volume and myocardial mass throughout the cardiac cycle in order to compute LV systolic and diastolic function. The full protocol has been described in detail previously.^26^ LVH was defined as LV mass/ Body surface area (BSA)> 88 g/m^2^ in women and > 102 g/m^2^ in men.^27^ Diastolic function was assessed using the LV volume‐filling time course.^28^, ^29^ The following CMR parameters were used to assess diastolic function:

1. Peak filling rate (PFR): Calculated as the maximal change in LV volume between sequential temporal phases (Δ volume/Δ time). The PFR was adjusted for stroke volume to generate normalized peak filling rate. The time needed from end‐systole to reach this was recorded (time to PFR).^29^
2. Diastolic volume recovery: The time in diastole required for recovery of 80% of stroke volume (DVR 80).^29^
3. Left atrial volume indexed by body surface area (LAVI)
4. E and A waves maximum velocity: Velocity encoded, phase contrast MRI was used to measure early (E) and late (A) diastolic mitral valve inflow velocities by drawing LV inflow contours and measuring flow throughout the cardiac cycle^30^

Kidney function was measured using estimated glomerular filtration rate (eGFR) from serum creatinine levels using the 2021 CKD‐EPI formula, adjusted for BSA.^31^

### 2.1 Statistical analysis

Descriptive analysis was performed to describe the variables of interest. Continuous variables were presented as mean ± standard deviation (std) or mean and interquartile range (IQR) based on Shapiro‐Wilk normality test and were compared between controlled RHTN and CHTN patients using two sample t‐test or Mann Whitney test, respectively. Categorical variables were presented as frequencies and percentages and were compared between the two groups using chi‐squared test of Fisher's exact test. *P* value of < .05 was used to determine significance. All analyses were performed using SPSS 24.0 (SPSS, Inc, Chicago, Illinois, USA).

## 3 RESULTS

A total of 182 patients met the inclusion criteria for the study. After undergoing 24‐hr ABPM and CMR, the final cohort included 69 controlled RHTN patients and 63 CHTN patients (Figure 2). There were no differences between groups in age, ethnicity, gender, smoking status or dyslipidemia, except for a higher prevalence of diabetes mellitus in the controlled RHTN group. Prevalence of comorbidities such as coronary artery disease, heart failure, arrhythmia, previous stroke/transient ischemic attack and peripheral arterial disease was similar in the two groups. Both groups had high body mass index (BMI) (33 ± 5.2 vs 32.6 ± 7 kg/m^2^; *P* = .731) and a high prevalence of dyslipidemia (70% vs 62%) in the controlled RHTN and CHTN groups, respectively. Clinic and 24‐h ABPM BPs (both systolic and diastolic) were similar in both groups. The median number of anti‐hypertensive medications was 4 (IQR 4–5) and 3 (IQR2+3) in the controlled RHTN and CHTN groups, respectively (*P* < .001). Patients with controlled RHTN were more likely to be on a thiazide diuretic, mineralocorticoid receptor antagonist (MRA), calcium channel blocker, combined α‐β blocker or central acting α2 agonist (Table 1).

**TABLE 1:** Anti‐hypertensive medications taken

	controlled RHTN (No. = 69)	CHTN (No. = 63)	*P*
Angiotensin‐converting enzyme inhibitors	35 (50.7)	22 (34.9)	.067
Angiotensinogen receptor blockers	28 (40.6)	28 (44.4)	.654
Calcium channel blockers	55 (79.7)	37 (58.7)	.009
Thiazide diuretics	62 (89.9)	38 (60.3)	<.001
Loop diuretics	4 (5.8)	0	.121
Amiloride & Triamterene	1 (1.4)	2 (3.2)	.606
Mineralocorticoid receptor antagonists	46 (66.7)	4 (6.3)	<.001
α‐blockers	4 (5.8)	0	.121
β‐blockers	26 (37.7)	14 (22.2)	.054
Combined α‐β blockers	18 (26.1)	4 (6.3)	.002
Central acting α2 agonists	17 (24.6)	1 (1.6)	<.001
Nitrates	3 (4.3)	1 (1.6)	.621
Potassium channel opener	1 (1.4)	0	1.000
Total antihypertensive medications	4 (4‐5)	3 (2‐3)	<.001

Data is presented as number of patients (percentage) or median (IQR).

Patients with controlled RHTN had higher LVMI compared to patients with CHTN (64.4 ± 22.5 vs 56.9 ± 11.5; *P* = .017). LV stroke volume indexed by BSA, ejection fraction, LV end‐diastolic volumes indexed by BSA (LVEDVI), and LV end‐systolic volumes indexed by BSA (LVESVI) were all similar in the two groups (Tables 2 and 3). Diastolic function parameters, including E:A ratio, proportion of patients with E:A ratio ≤.8 or ≥2, PFR, time to PFR, normalized PFR, DVR 80, percentage of diastole needed to reach DVR 80 and LAVI were similar in the two groups (Table 3). LV stroke volume/pulse pressure was similar in both groups, regardless whether it was assessed based on clinic or 24‐h ABPM readings (Table 3).

**TABLE 2:** Cardiac anatomical parameters based on CMR

MRI characteristics	controlled RHTN (No. = 69)	CHTN (No. = 63)	*P*
***Left ventricle***			
LVM (g)	135.5 ± 55.7	118.2 ± 32.8	.031
LVMI (g/m^2^)	64.4 ± 22.5	56.9 ± 11.5	.017
Left ventricle end‐systolic volume (mL)	50.2 ± 18.3	54.7 ± 25.4	.244
Left ventricle end‐systolic volume indexed by BSA (mL/m^2^)	23.7 ± 8.1	25.8 ± 11.9	.224
Left ventricle end‐diastolic volume (mL)	141.2 ± 36.4	139.9 ± 37.5	.844
Left ventricle end‐diastolic volume indexed by BSA (mL/m^2^)	66.5 ± 15.1	66.1 ± 18.1	.907
Left ventricle posterior wall thickness (mm)	8.5 ± 2.2	7.6 ± 1.5	.005
Inter ventricular septum thickness (mm)	11.9 ± 3.1	11.0 ± 2.1	.085
***Left atrium***			
Left atrium volume (mL)	65.4 ± 24.2	64 ± 21.8	.735

LVESVI and LVEDVI values are missing for one patient from each group.

Abbreviations: BSA, body surface area; LVM, Left ventricular mass; LVMI, Left ventricular mass index.

**TABLE 3:** Cardiac systolic and diastolic function

MRI characteristics	Controlled RHTN (No. = 69)	CHTN (No. = 63)	*P*
***Left Ventricular Systolic function***			
LVSV (mL)	90.9 ± 25.5	85.0 ± 20.1	.143
LVSV indexed by BSA	42.8 ± 10.6	40.2 ± 10.2	.159
Left ventricle ejection fraction (%)	64.7 ± 7.8	62.4 ± 8.4	.113
LVSV/pulse pressure clinic	2.0 ± .7	1.98 ± .7	.792
LVSV/pulse pressure ABPM	1.5 ± .5	1.5 ± .5	.844
***Left Ventricular Diastolic function***			
DVR 80 (ms)	481.1 ± 139.6	450.7 ± 123.6	.189
Percentage of diastole needed for DVR 80 (%)	88.0 ± 13.5	89.0 ± 8.8	.629
Left ventricle PFR (mL/s)	481.0 ± 183.8	481.6 ± 205.6	.987
Time to PFR (ms)	447.5 ± 199.6	449.1 ± 160.5	.960
PFR normalized to stroke volume (s)	5.6 ± 1.6	5.5 ± 1.4	.869
% Diastole to reach PFR	82.1 ± 28.7	88.5 ± 20.7	.138
B‐type Natriuretic Peptide	41.4 ± 55.8	31.6 ± 32.4	.222
LAV indexed by body surface area (mL/m2)	31.5 ± 10.9	31.2 ± 9.4	.861
E:A ratio	1.13 ± .5	1.19 ± .5	.541
E:A ratio ≤.8	12 (20.3)	8 (14.0)	.369
E:A ratio ≥2	2 (3.4)	4 (7.0)	.378

*Note*: Data is presented as mean ± standard deviation or presence of trait (percentage).

BNP value was missing for five patients in the CHTN group and two in the RHTN group, LVSVI values are missing for one patient from each group. E‐A ratio could not be calculated for 10 controlled RHTN and 6 CHTN patients due to absent A‐wave due to atrial fibrillation or lack of mitral flow data.

Abbreviations: BSA, body surface area; DVR 80, diastolic volume recovery of 80% of stroke volume; LAV, left atrial volume; LVSV, Left ventricle stroke volume; PFR, peak filling rate.

### 3.1 Masked uncontrolled versus true controlled HTN

A separate analysis was performed to compare characteristics between true controlled hypertensive patients (have controlled BP in clinic and on 24‐h ABPM) and masked uncontrolled patients of both phenotypes, RHTN and non‐RHTN. The only demographic differences found were higher BMI in masked uncontrolled RHTN (MRHTN) patients compared to true controlled RHTN patients (34.4 ± 4.7 vs 31.4 ± 5.4 kg/m^2^; *P* = .019) and higher prevalence of diabetes mellitus in masked uncontrolled RHTN compared to controlled RHTN (20 (54.1%) versus 9 (28.1%); *P* = .03) (Supplementary Table S1).

Masked uncontrolled hypertensive patients of both phenotypes (resistant and non‐resistant) had higher LVM than their counterparts with true controlled HTN, LVMI was also higher in MRHTN compared to controlled RHTN (Supplementary Table S2). However, patients with true controlled RHTN and true CHTN groups had similar LVM and LVMI (Supplementary Table S3). Four patients in the RHTN group had LVH, all of them had MRHTN, none of the patients with non‐RHTN had LVH. Diastolic function parameters, namely PFR, time to PFR, normalized PFR, DVR 80, percentage of diastole needed to reach DVR 80 and LAVI were all similar in true controlled RHTN compared to masked uncontrolled RHTN patients and in true CHTH patients compared to masked uncontrolled non‐RHTN patients (MUCH) (Supplementary Table S4).

Patients with controlled RHTN had higher pulse pressure than those with CHTN on 24‐h ABPM, but not on clinic BP (Table 4). On the other hand, patients with true controlled RHTN and true CHTN had similar pulse pressure (Supplementary Table S3). Patients with MUCH had higher pulse pressure than those with true CHTN on both clinic BP and 24‐h ABPM. Patients with MRHTN had higher pulse pressure then those with true controlled RHTN on 24‐h ABPM but not clinic BP (Supplementary Table S1). Patients with MRHTN had similar pulse pressure to those with MUCH on both clinic and 24‐h ABPM (data not shown).

**TABLE 4:** Patient characteristics

Characteristics	controlled RHTN (No. = 69)	CHTN (No. = 63)	*P*
***Demographics***			
Age (years)	58.4 ± 11.5	60.3 ± 10.5	.334
BMI (kg/m^2^)	33 ± 5.2	32.6 ± 7	.731
Males	36 (52.2)	37 (58.7)	.449
African Americans	38 (55.1)	24 (38.1)	.051
Current smoker	3 (4.3)	7 (11.1)	.142
Dyslipidemia	48 (69.6)	39 (61.9)	.354
Type 2 diabetes	29 (42)	10 (15.9)	.001
Congestive heart failure	6 (8.7)	2 (3.2)	.184
Arrhythmia	6 (8.7)	4 (6.3)	.611
Coronary artery disease	11 (15.9)	8 (12.7)	.596
Peripheral Vascular Disease	5 (7.2)	3 (4.8)	.550
Previous stroke/transient ischemic attack	10 (14.5)	6 (9.5)	.382
***Blood pressure parameters***			
Clinic systolic BP (mmHg)	118.1 ± 10.2	117.2 ± 9.3	.586
Clinic diastolic BP (mmHg)	71.3 ± 8.8	73.2 ± 7.2	.174
Clinic heart rate (beats/min)	70.3 ± 11.1	74.2 ± 12.6	.059
ABPM overall systolic BP (mmHg)	135 ± 15.7	131.2 ± 13.3	.133
ABPM overall diastolic BP (mmHg)	73.6 ± 9.8	74.5 ± 8.6	.578
ABPM overall heart rate (beats/min)	70.9 ± 11.5	73.5 ± 12	.207
ABPM daytime systolic BP (mmHg)	137.7 ± 15.3	133.7 ± 13.6	.114
ABPM daytime diastolic BP (mmHg)	75.7 ± 9.7	76.6 ± 8.8	.569
ABPM daytime heart rate (beats/minute)	72.3 ± 12	75.1 ± 12.1	.185
Non dipping nocturnal BP	30 (43.5)	29 (46)	.768
Clinic pulse pressure (mm Hg)	46.8 ± 10.3	44.0 ± 9.2	.098
24‐h‐ ABPM pulse pressure (mm Hg)	61.5 ± 12.1	56.8 ± 10.8	.020
***GFR***			
Estimated GFR based on serum Cr (mL/min/1.73 m^2^)	56.8 ± 25.3	72.6 ± 26.5	.004

*Note*: Data is presented as mean ± standard deviation or presence of trait (percentage).

Serum Cr levels were missing for 17 patients in the CHTN and 19 in the controlled RHTN group.

Abbreviations: ABPM, Ambulatory BP monitoring; BMI, Body mass index; Cr, Creatinine; GFR, Glomerular Filtration Rate.

There were no significant differences in the proportion of patients with non‐dipping nocturnal BP per 24‐h ABPM in all groups, regardless of whether patients were assessed as two groups, or four with masked uncontrolled HTN assessed separately (Table 4 and Supplementary Table S1).

### 3.2 Analyses based on age, dyslipidemia, and diabetes

Patients from the four groups (true controlled RHTN, true CHTN, MRHTN, and MUCH) were combined and had their diastolic function analyzed based on age, presence of dyslipidemia and diabetes mellitus. The time to reach PFR and percentage of diastole needed to reach PFR were higher in patients with older age (≥ 61 years) compared to those 60 years or younger (median age of the patient population used for cutoff) (487.8 ± 167.7 vs 403.7 ± 187.1 ms; *P* = .007 and 90.0% ± 21.1% vs 79.7% ± 28.5%; *P* = .019, respectively). Time to reach PFR and percentage of diastole needed to reach PFR were also higher in patients with dyslipidemia compared to those without (474.1 ± 179.6 vs 398.3 ± 176.0 ms; *P* = .022 and 88.7% ± 23.4% vs 78.3% ± 27.7%; *P* = .034, respectively) (Supplementary Table S5). Linear regression modelling showed that the latter association was independent of age, gender, and BMI.

### 3.3 Kidney function

Patients with controlled RHTN had lower estimated GFR based on serum creatinine than those with CHTN (56.8 ± 25.3 vs 72.6 ± 26.5 mL/min/1.73m^2^; *P* = .004).

## 4 DISCUSSION

### 4.1 Cardiac structure and volumes

Patients with controlled RHTN had higher LVMI than those with CHTN, however that was likely driven by the patients with MRTHN as shown in the separate groups sub‐analysis (Supplementary Tables 2–4). In addition, this study is the first to demonstrate that cardiac structure and function were similar in true controlled RHTN patients compared to those with true controlled non‐RHTN. The similar LVMI in both groups may be partially attributed to the frequent use of MRAs in the controlled RHTN group. Use of an MRA such as spironolactone or eplerenone has been shown to reverse the LVH in patients with RHTN.^32^, ^33^ The lack of LVH in the true controlled RHTN and true CHTN can be attributed to intensive BP control and the similar cardiac volumes in these groups.

Although increased cardiac volumes have been reported in RHTN patients, indicating volume overload, we did not find any differences in LVEDVI, LVESVI, Left atrial volumes or BNP levels between patients with controlled RHTN and CHTN regardless of the inclusion of patients with masked uncontrolled HTN. Since patients with controlled RHTN were more likely to be prescribed thiazide diuretics and MRAs, this suggests that use of these agents facilitates achieving BP control and results in a volume status in individuals with controlled RHTN similar to that in patients with non‐resistant hypertension. LV stroke volume/pulse pressure ratio was similar in patients with controlled RHTN versus CHTN, this parameter has been associated with arterial compliance, CV death, LV concentric geometry and eccentric LV hypertrophy in patients with HTN.^34^

### 4.2 Cardiac diastolic function

The similar diastolic function seen in all groups may be related to sustained BP control. Many factors other than HTN are known to increase the risk of diastolic dysfunction, such as older age, obesity, coronary artery disease, history of myocardial infarction and systolic dysfunction, were similar in both groups, suggesting that they were not confounders.^12^, ^13^ MRA use in the controlled RHTN group could also have improved diastolic function. In this patient population 67% of patients with controlled RHTN were taking an MRA (Table 1). Spironolactone has been shown to improve diastolic function, especially in patients with LVH and heart failure with preserved ejection fraction (HFpEF).^35^, ^36^, ^37^ E/e’, LA area, E:A ratio and deceleration time all showed significant changes indicating improvement in diastolic function by echocardiogram. However, it is worth noting that a study from our center that included patients with RHTN did not show improvement in diastolic function assessed via CMR‐measured E:A ratios with use of spironolactone.^38^ That study, however, defined RHTN as clinic BP > 140/90 mmHg (according to the guidelines’ definition of HTN at that time) and compared diastolic function in patients with RHTN both before and after 6 months of treatment with spironolactone. Further, unlike our study, there was no CHTN comparator group.

Patients with older age and dyslipidemia had longer time to PRF and percentage of diastole needed to reach PRF, correlating with worse diastolic function regardless of their HTN phenotype. Age is a well‐known risk factor for diastolic dysfunction.^12^, ^13^ Dyslipidemia, however, has been reported as a risk factor in only a few studies.^39^, ^40^, ^41^ The effects of dyslipidemia and aging were likely more pronounced in our study, given that the patients had similar blood pressure, one of the cardinal risk factors for diastolic dysfunction. The association between dyslipidemia and the PFR parameters was independent of age, gender or BMI.

This association is likely due to the effects of dyslipidemia on the myocardium. High cholesterol diet has been linked to impaired cardiac relaxation due to alterations in cellular calcium currents in rabbit models.^39^ Low HDL levels were also associated with higher E:A ratios on echocardiography in humans.^40^ Another study found that high triglyceride levels were positively correlated with the E:A ratios on echocardiography.^41^ The latter two studies included hypertensive patients not taking any anti‐hypertensive medications from China and patients with metabolic syndrome, most of which had HTN, from the United States respectively. Our study included patients with HTN on anti‐hypertensive medications with well controlled BP based on multiple clinic readings and assessed diastolic function using CMR, which has less user‐dependent variability than echocardiography. This lowers the confounding effect of other variables that might affect diastolic function.

### 4.3 Patients with masked uncontrolled HTN

To the best of our knowledge, this is the first study to assess cardiac structure and function in patients with MRHTN. Patients with MRHTN and masked uncontrolled non‐RHTN had higher LVM and LVMI compared to those with true controlled RHTN or CHTN, respectively. This is in line with data from a previous study showing that patients with masked hypertension not taking any anti‐hypertensive medications had higher LVM and LVMI than normotensive patients.^42^ Our study is novel in that the patients in this study were on medications and the study population included patients with RHTN. Furthermore, CMR was used here, while echocardiography was used in the previous study. All patients with LVH were in the MRHTN group, likely due to the prolonged course of hypertension, often not well controlled, usually seen in patients with RHTN. Importantly these patients were shown to have masked uncontrolled HTN on ABPM even when the clinic BP was well controlled on 3 consecutive visits. The current data show that patients with masked uncontrolled HTN, whether RHTN or non‐RHTN, have diastolic function similar to those with controlled RHTN and non‐RHTN. However, it is important to note that our data may be limited, as ABPM was assessed only once and the BP values during the study do not necessarily reflect sustained controlled or uncontrolled BP throughout the decades of HTN in these patients.

The wider pulse pressure seen in this study is likely due to patients with masked uncontrolled HTN as seen in the subgroup analyses (Supplementary Table S1). This difference in pulse pressure has also been shown in previous data from our group. The etiology of pulse pressure widening is not fully understood, but may be due to increased sympathetic tone, as reflected in higher out‐of‐clinic catecholamines and metanephrines on 24‐h urine collections.^43^ Although the method of calculating non‐dipping nocturnal BP is not well defined, it has been shown to be associated with more adverse target organ damage in other studies.^44^ It is important to note that, given the relatively low and well controlled BP in the participants in this study, non‐dipping is more common in this study population likely due to their systolic and diastolic pressures being closer to the physiologic norms. The prevalence was accordingly relatively high in all groups. There is an association of non‐dipping BP with LVH, but it was shown in patients which would be defined as having poorly controlled BP according to recent guidelines.^25^ In addition, the association of non‐dipping BP with adverse outcomes was nonsignificant in many studies when adjusted to nighttime and 24‐h BP.^45^, ^46^ This shows that non‐dipping is unlikely to be a major factor of cardiac remodeling in our patient population which has well controlled BP and low nocturnal BP.

### 4.4 Study design

The observation that 55.4% and 48.1% of those with three consecutive controlled clinic BP readings had masked HTN on ABPM affirms the importance of using ABPM or home BP monitoring to diagnose masked HTN, even in patients with consistently controlled BP on multiple clinic visits. Thresholds for BP control using average daytime ABPM (≤135/85 mmHg) and the definition of valid ABPM readings (> 80%) were based on the scientific statements and expert opinion that were operative when the study was designed,^23^, ^24^ However the recent ACC/AHA HTN guidelines now define controlled BP on average daytime ABPM readings as ≤130/80 mmHg,^47^ and the AHA statement on measurement of BP recommends following the Canadian and European guidelines in requiring at least 70% of the readings to be valid for the definition of complete ABPM.^18^

The strengths of the current study include enrolling patients after three consecutive controlled clinic BP readings obtained unattended and according to the AHA HTN measurement recommendations, use of ABPM to identify patients with masked uncontrolled hypertension, and use of CMR, which is considered the current gold standard for studying the structure and function of the heart. Limitations include the small sample size and the cross‐sectional design, which does not account for changes in a baseline cardiac structure and function over time. Some participants were followed for only a short time at our institution prior to the beginning of the study, making it difficult to establish the duration of HTN and of BP control. Lastly most patients in the study, even in the masked uncontrolled groups, had well controlled BP. This limits the external validity of the study in patients with higher BP treatment targets, such as elderly and frail patients. Further prospective studies investigating both the prevention and reversal of cardiac remodeling and assessment of kidney function are needed to validate the results of our study.

## 5 CONCLUSION

This study shows that patients with controlled RHTN have higher LVMI, likely driven by patients with MRHTN and have diastolic function comparable to those of patients with CHTN. This suggests that intensive treatment and control of BP in and out of clinic may result in improved cardiac structure and function in RHTN patients. Patients with dyslipidemia had diastolic filling patterns suggestive of diastolic dysfunction regardless of their BP control.

## AUTHOR CONTRIBUTIONS

Faris MATANES, Data analysis, writing the manuscript; Mohammed SIDDIQUI, patient enrollment, data analysis; Alejandro VELASCO, data analysis; Oleg SHARIFOV, Supervision and manuscript edits ; Eric KREPS, data analysis; Tanja DUDENBOSTEL, Supervision and manuscript edits; Eric K JUDD, Supervision and manuscript edits; Bin ZHANG, Biostatistical analysis ; Steven G LLOYD, Supervision and manuscript edits; Suzanne OPARIL, Supervision and manuscript edits, physician involved in patients’ care ; David A CALHOUN, Supervision and manuscript edits, physician involved in patients’ care and PI of the NIH grant

## CONFLICT OF INTEREST

All authors declare no conflict of interest.

## Supporting information

Supporting Information
