# Supplementary material for: Adverse cardiac remodeling is absent in patients with true controlled resistant hypertension
Source: J Clin Hypertens (Greenwich). 2023 Apr 3;25(5):416–25. doi: 10.1111/jch.14625 (PMC10184488; doi:10.1111/jch.14625)
Supplement: Supplementary file 1 — Supporting Information [file JCH-25-416-s001.docx]

| Supplementary Table 1. Demographic Characteristics of Patients with Masked uncontrolled Vs. True Controlled HTN | | | | | | |
| --- | --- | --- | --- | --- | --- | --- |
| Characteristics | True controlled RHTN (n=32) | MRHTN (n=37) | P Value | True CHTN (n=34) | MUCH (n=29) | P Value |
| Age (years) | 61.3±11.5 | 56.8 ± 11.0 | 0.095 | 60.6 ± 11.2 | 60.9 ± 10.0 | 0.913 |
| BMI (Kg/m^2^) | 31.4 ± 5.4 | 34.4 ± 4.7 | 0.019 | 32.3 ± 7.4 | 33.1 ± 6.6 | 0.670 |
| Males | 19 (59.4) | 17 (45.9) | 0.265 | 18(52.9) | 19 (65.5) | 0.312 |
| African Americans | 17 (53.1) | 21 (56.8) | 0.762 | 14(41.2) | 10(34.5) | 0.586 |
| Current smoker | 1 (3.1) | 2 (5.4) | 1.000 | 2 (5.9) | 5 (17.2) | 0.233 |
| Dyslipidemia | 24 (75) | 24 (64.9) | 0.362 | 19 (55.9) | 20 (69) | 0.287 |
| Type 2 diabetes | 9 (28.1) | 20 (54.1) | 0.030 | 3 (8.8) | 7 (24.1) | 0.165 |
| Congestive heart failure | 30(93.8) | 33(89.2) | 0.503 | 1(2.9) | 1(3.4) | 1.000 |
| Arrhythmia | 27(84.4) | 36(97.3) | 0.057 | 3(8.8) | 1(3.4) | 0.618 |
| Coronary artery disease | 26(81.3) | 32(86.5) | 0.553 | 5(14.7) | 3(10.3) | 0.716 |
| Peripheral Vascular Disease | 31(96.9) | 33(89.2) | 0.219 | 3(8.8) | 0(0.0) | 0.243 |
| Previous stroke/transient ischemic attack | 3 (9.4) | 7 (18.9) | 0.320 | 3 (8.8) | 3 (10.3) | 1.000 |
| ABPM overall systolic BP (mmHg) | 122.2 ± 7.1 | 146.2 ± 12.2 | <0.0001 | 121.8 ± 6.8 | 142.2 ± 10.3 | <0.0001 |
| ABPM overall diastolic BP (mmHg) | 67.6 ± 6.5 | 78.8 ± 9.2 | <0.0001 | 70.4 ± 7.1 | 79.2 ± 7.8 | <0.0001 |
| ABPM overall heart rate (beats/minute) | 68.8 ± 10.8 | 72.8 ± 11.9 | 0.16 | 72.6 ± 12.0 | 74.7 ± 12.2 | 0.501 |
| ABPM daytime systolic BP (mmHg) | 124.6 ± 7.0 | 149.1 ± 10.8 | <0.0001 | 124.0 ± 6.9 | 145.1 ± 10.2 | <0.0001 |
| ABPM daytime diastolic BP (mmHg) | 69.4 ± 6.7 | 81.1 ± 8.5 | <0.0001 | 72.6 ± 7.6 | 81.3 ± 7.7 | <0.0001 |
| ABPM daytime heart rate (beats/minute) | 70.0 ± 11.6 | 74.2 ± 12.2 | 0.151 | 74.4 ± 12.3 | 75.9 ± 12.1 | 0.64 |
| Non-dipping nocturnal BP | 14 (43.8) | 16 (43.2) | 0.966 | 16 (47.1) | 13 (44.8) | 0.859 |
| Clinic Pulse pressure (mm Hg) | 45.6 ± 10.7 | 47.9 ± 10 | 0.353 | 41.6 ± 7.9 | 46.7 ± 9.8 | 0.027 |
| 24-hour ABPM pulse pressure (mm Hg) | 54.7 ± 7.4 | 67.4 ± 12.4 | <0.001 | 51.3 ± 8.8 | 63.2 ± 9.3 | <0.001 |

MRHTN indicates masked uncontrolled resistant hypertension; MUCH, masked uncontrolled non- resistant hypertension; BMI, body mass index; CHF, Congestive heart failure; CAD, coronary artery disease; PVD, peripheral vascular disease; TIA, transient ischemic attack; ABPM, ambulatory blood pressure monitoring

Supplementary Table 2. Cardiac Anatomical Parameters of Patients with Masked uncontrolled Vs. True Controlled HTN based on CMR

| MRI characteristics | True cRHTN (n=32) | MRHTN (n=37) | P Value | True CHTN (n=34) | MUCH (n=29) | P Value |
| --- | --- | --- | --- | --- | --- | --- |
| ***Left ventricle*** |  |  |  |  |  |  |
| LVM (g) | 118.5 ± 38.7 | 150.6 ± 64.0 | 0.016 | 110.6 ± 28.8 | 127.5 ± 35.3 | 0.043 |
| LVMI (g/m2) | 56.6 ± 13.2 | 70.3 ± 26.7 | 0.007 | 54.4 ± 10.3 | 59.2 ± 12.2 | 0.093 |
| Left ventricle end-systolic  volume (mL) | 51.5 ±  19.6 | 49.1 ± 17.2 | 0.595 | 53.3 ± 27.7 | 56.5 ± 22.7 | 0.630 |
| Left ventricle end-diastolic  volume (mL) | 139.5 ± 34.2 | 142.7 ± 38.7 | 0.726 | 138.4 ± 37.6 | 141.7 ± 38.1 | 0.732 |
| Left ventricle posterior wall  thickness (mm) | 8.0 ± 1.7 | 9.0 ± 2.5 | 0.065 | 7.2 ± 1.4 | 8.0 ± 1.5 | 0.093 |
| Inter ventricular septum  thickness (mm) | 11.1 ± 2.6 | 12.5 ± 3.4 | 0.068 | 10.5 ± 2.1 | 11.7 ± 2.1 | 0.022 |
| ***Left atrium*** |  |  |  |  |  |  |
| Left atrium volume (mL) | 69.4 ± 27.7 | 61.8 ± 20.5 | 0.200 | 66.1 ± 22.9 | 61.2 ± 20.4 | 0.394 |

Supplementary Table 3. Data Comparing Patients with True Controlled RHTN Vs. true CHTN

| MRI characteristics | True controlled RHTN (n=32) | | True CHTN (n=34) | P value |
| --- | --- | --- | --- | --- |
| ***Cardiac Structure*** | | | | |
| LVMI | | 56.6 ± 13.2 | 54.4 ± 10.3 | 0.450 |
| ***Left Ventricular Systolic function*** | | | | |
| Left ventricle stroke  volume (mL) | 87.7 ± 21.9 | | 84.7 ± 20.4 | 0.570 |
| Left ventricle ejection  fraction (%) | 63.6 ± 7.9 | | 62.7 ± 8.5 | 0.657 |
| ***Left Ventricular Diastolic function*** | | | | |
| Time to recover 80% of LV stroke volume (DVR 80) (ms) | 485.5 ± 136.7 | | 475.8± 137.2 | 0.775 |
| Percentage of diastole needed for DVR 80, % | 87.2 ± 14.0 | | 89.2 ± 7.0 | 0.486 |
| Left ventricle peak filling  Rate (PFR) (mL/s) | 473.4 ± 174.8 | | 462.5 ± 206.5 | 0.818 |
| Time to PFR (ms) | 437.9 ± 203.4 | | 463.7 ± 180.4 | 0.587 |
| PFR normalized to stroke volume (s) | 5.6 ± 1.7 | | 5.5 ± 1.3 | 0.811+ |
| % diastole to reach PFR | 79.6 ± 31.7 | | 87.1 ± 22.3 | 0.272 |
| B-type Natriuretic Peptide (BNP) | 45.8 ± 66.8 | | 38.9 ± 38.9 | 0.619 |
| Left atrial volume indexed by body surface area (LAVI) (mL/m2) | 33.8 ± 12.2 | | 32.8 ± 9.4 | 0.705 |
| LAVI >34 | 15 (46.9%) | | 11 (32.4%) | 0.228 |
| Left atrial volume (mL) | 69.4 ± 27.7 | | 66.1 ± 22.9 |  |
| Early diastolic mitral inflow  maximal velocity (E) (cm/s) | 45.0 ± 13.5 | | 49.4 ± 12.4 | 0.178 |
| Late diastolic mitral inflow  maximal velocity (A) (cm/s) | 42.0 ± 11.7 | | 44.0 ± 12.2 | 0.515 |
| E/A ratio maximal velocity  Ratio | 1.19 ± 0.7 | | 1.21 ± 0.5 | 0.862 |
| Clinic Pulse pressure (mm Hg) | 45.6 ± 10.7 | | 41.6 ± 7.9 | 0.094 |
| 24-hour ABPM pulse pressure (mm Hg) | 54.7 ± 7.4 | | 51.3 ± 8.8 | 0.096 |

*A-wave was not present in one patient due to atrial fibrillation

**BNP was missing for 2 patients in the CHTN group and 1 in the controlled RHTN group

| Supplementary Table 4. Cardiac Systolic and Diastolic Function in Patients with Masked uncontrolled Vs. True Controlled HTN based on CMR | | | | | | |
| --- | --- | --- | --- | --- | --- | --- |
| MRI characteristics | True cRHTN  (n= 32) | MRHTN  (n= 37) | P Value | True CHTN  (n= 34) | MUCH  (n= 29) | P Value |
| ***Left Ventricular Systolic function*** |  |  |  |  |  |  |
| Left ventricle stroke  volume (mL) | 87.7 ± 21.9 | 93.8 ± 28.3 | 0.325 | 84.7 ± 20.4 | 85.3 ± 20.1 | 0.911 |
| Left ventricle ejection  fraction (%) | 63.6 ± 7.9 | 65.7 ± 7.6 | 0.279 | 62.7 ± 8.5 | 62.1 ± 8.5 | 0.785 |
| ***Left Ventricular Diastolic function*** |  |  |  |  |  |  |
| Time to recover 80% of LV stroke volume (DVR 80) (ms) | 485.5 ± 136.7 | 481.2 ± 142.5 | 0.898 | 467.2 ± 136.5 | 431.2 ± 105.63 | 0.253 |
| Percentage of diastole needed for DVR 80 (%) | 87.2 ± 14.0 | 89.6 ± 10.6 | 0.432 | 88.4 ± 10.4 | 89.7 ± 6.5 | 0.550 |
| Left ventricle peak filling  Rate (PFR) (mL/s) | 473.4 ± 174.8 | 487.6 ± 193.4 | 0.752 | 462.5 ± 206.5 | 504.0 ± 205.9 | 0.429 |
| Time to PFR (ms) | 437.9 ± 203.4 | 455.9 ± 198.8 | 0.712 | 463.7 ± 180.4 | 432.0 ± 134.6 | 0.438 |
| PFR normalized to stroke volume (s) | 5.6 ± 1.7 | 5.6 ± 1.7 | 0.879 | 5.5 ± 1.3 | 5.6 ± 1.6 | 0.950 |
| % diastole to reach PFR | 81.4 ± 30.5 | 84.2 ± 26.0 | 0.685 | 87.1 ± 22.3 | 90.2 ± 18.9 | 0.564 |
| B-type Natriuretic Peptide (BNP) | 45.8 ± 66.8 | 37.7 ± 44.8 | 0.561 | 38.9 ± 38.9 | 22.6 ± 19.0 | 0.055 |
| Left atrial volume indexed by body surface area (LAVi) (mL/m2) | 33.8 ± 12.2 | 29.5 ± 9.3 | 0.106 | 32.8 ± 9.4 | 29.2 ± 9.1 | 0.146 |
| Left atrial volume (mL) | 69.4 ± 27.7 | 61.8 ± 20.5 | 0.200 | 66.1 ± 22.9 | 61.2 ± 20.4 | 0.394 |

Supplementary Table 5. Diastolic Function in all Patients Based on Age, Dyslipidemia and Diabetes Mellitus

|  | Age | | P value | Dyslipidemia | | P value | DM | | P value |
| --- | --- | --- | --- | --- | --- | --- | --- | --- | --- |
|  | ≤60  (n= 62) | >60  (n= 70) |  | Patients without trait  (n= 45) | Patients with trait  (n= 87) |  | Patients without trait  (n= 93) | Patients with trait (n=39) |  |
| Left ventricle end-diastolic  volume (mL) | 145.9 ± 38.8 | 132.2 ± 34.9 | 0.034 | 135.9 ± 42.8 | 140.0 ± 34.2 | 0.550 | 140.1 ± 39.1 | 135.2 ± 32.7 | 0.489 |
| Time to recover 80% of LV stroke volume (DVR 80) (ms) | 448.2 ± 131.1 | 482.8 ± 132.8 | 0.136 | 441.9 ±117.7 | 479.3 ± 138.6 | 0.125 | 471.3 ± 132.1 | 455.2 ± 134.9 | 0.524 |
| Percentage of diastole needed for DVR 80 (%) | 88.4 ± 11.0 | 88.6 ± 11.9 | 0.890 | 86.5 ±12.7 | 89.5 ±10.6 | 0.150 | 88.3 ± 11.9 | 88.9 ± 10.5 | 0.791 |
| Patients with a percentage of diastole for DVR>77% | 60 (96.8%) | 64 (91.4%) | 0.199 | 41 (91.1%) | 83 (95.4%) | 0.327 | 88 (94.6%) | 36 (92.3%) | 0.611 |
| Left ventricle peak filling  Rate (PFR) (mL/s) | 515.4 ± 202.7 | 451.1 ± 181.6 | 0.057 | 452.5 ± 187.8 | 496.2 ± 196.2 | 0.221 | 467.0 ± 179.2 | 515.3 ± 223.7 | 0.193 |
| PFR >344 | 53 (85.5%) | 45 (64.3%) | 0.005 | 30 (66.7%) | 68 (78.2%) | 0.152 | 67 (72.0%) | 31 (79.5%) | 0.372 |
| Time to PFR (ms) | 403.7 ± 187.1 | 487.8 ± 167.7 | 0.007 | 398.3 ± 176.0 | 474.1 ± 179.6 | 0.022 | 449.5 ± 182.7 | 445.3 ± 180.3 | 0.904 |
| PFR normalized to stroke volume (s) | 5.6 ± 1.4 | 5.6 ± 1.7 | 0.978 | 5.4 ± 1.5 | 5.6 ± 1.6 | 0.540 | 5.4 ± 1.4 | 6.0 ± 1.8 | 0.077 |
| Patients with normalized PFR >4.02s | 56 (90.3%) | 60 (85.7%) | 0.418 | 37 (82.2%) | 79 (90.8%) | 0.152 | 80 (86.0%) | 36 (92.3%) | 0.313 |
| % Diastole to reach PFR | 79.7 ± 28.5 | 90.0 ± 21.1 | 0.019 | 78.3 ± 27.7 | 88.7 ± 23.4 | 0.034 | 84.18 ± 25.986 | 87.45 ± 23.770 | 0.501 |
| 100% needed | 36 (58.1%) | 45 (64.3%) | 0.464 | 22 (48.9%) | 59 (67.8%) | 0.034 | 56 (60.2%) | 25 (64.1%) | 0.676 |
| B-type Natriuretic Peptide (BNP) | 24.3 ± 36.1 | 48.4 ± 52.0 | 0.003 | 37.1 ± 44.8 | 36.8 ± 47.5 | 0.974 | 37.1 ± 48.7 | 36.2 ± 41.4 | 0.918 |
| Left atrial volume indexed by body surface area (LAVi) (mL/m2) | 31.3 ± 9.4 | 31.5 ± 10.9 | 0.889 | 31.2 ± 10.0 | 31.5 ± 10.3 | 0.878 | 31.7 ± 9.9 | 30.7 ± 10.9 | 0.595 |
| - Age threshold was set as 60 based on median age of the whole patient population | | | | | | | | | |
